# Twelve Weeks of Whole Body Vibration Training Improve Regucalcin, Body Composition and Physical Fitness in Postmenopausal Women: A Pilot Study

**DOI:** 10.3390/ijerph17113940

**Published:** 2020-06-02

**Authors:** Jorge Pérez-Gómez, José Carmelo Adsuar, Miguel Ángel García-Gordillo, Pilar Muñoz, Lidio Romo, Marcos Maynar, Narcis Gusi, Redondo P. C.

**Affiliations:** 1 HEME Research Group, University of Extremadura, 10003 Cáceres, Spain; carmelo.adsuar@gmail.com (J.C.A.); pilarmulopez92@gmail.com (P.M.); lidioromoc@gmail.com (L.R.); 2 Facultad de Administración y Negocios, Universidad Autónoma de Chile, sede Talca 3467987, Chile; miguelgarciagordillo@gmail.com; 3 Faculty of Sport Science, University of Extremadura, 10003 Cáceres, Spain; mmaynar@unex.es (M.M.); ngusi@unex.es (N.G.); 4 Department of Physiology, University of Extremadura, 10003 Cáceres, Spain; pcr7726@gmail.com

**Keywords:** exercise, human, physical conditioning, stability

## Abstract

(1) *Background*: Regucalcin or senescence marker protein 30 (SMP30) is a Ca^2+^ binding protein discovered in 1978 with multiple functions reported in the literature. However, the impact of exercise training on SMP30 in humans has not been analyzed. Aging is associated with many detrimental physiological changes that affect body composition, functional capacity, and balance. The present study aims to investigate the effects of whole body vibration (WBV) in postmenopausal women. (2) *Methods*: A total of 13 women (aged 54.3 ± 3.4 years) participated in the study. SMP30, body composition (fat mass, lean mass, and bone mass) and physical fitness (balance, time up and go (TUG) and 6-min walk test (6MWT)) were measured before and after the 12 weeks of WBV training. (3) *Results*: The WBV training program elicited a significant increase in SPM30 measured in plasma (27.7%, *p* = 0.004) and also in 6MWT (12.5%, *p* < 0.001). The WBV training also significantly reduced SPM30 measured in platelets (38.7%, *p* = 0.014), TUG (23.1%, *p* < 0.001) and total body fat mass (4.4%, *p* = 0.02). (4) *Conclusions*: There were no significant differences in balance, lean mass or bone mass. The present study suggests that 12 weeks of WBV has the potential to improve SPM30, fat mass, TUG and 6MWT in postmenopausal women.

## 1. Introduction

Regucalcin (SMP30) is a calcium binding protein discovered in 1978 [1], and it has been shown to be a downregulated protein in aging rats [2]. SMP30 has exhibited anti-tumor activity [3,4], and its expression in cancer is significantly higher compared to normal tissue [5]. SMP30 is also important for maintaining calcium homeostasis [6], preventing apoptosis [6], and for its anti-inflammatory and antioxidant functions [7]. Recently, SMP30 has been tied to osteoporosis [5], due to the studies done on bone metabolism in rats [8]. However, despite that the beneficial effect of exercise in bone mineralization has been reported [9], no studies have analyzed the effect of exercise on SMP30 in humans.

Fractures and obesity are major health concerns in the elderly. One-third of women, over the age of 50, suffer at least one fracture during their lifetime, with the consequences of morbidity and economic cost to the health system [10]. Falls are a common cause of fracture in the elderly [11]. Balance is the ability to maintain the centre of gravity over the base of support [12] and it deteriorates with aging [13]. Fallers shows worse balance than non-fallers [14]. Meanwhile, in western developed societies, obesity is a growing public health problem, majorly affecting elderly people [15]. Thus, exercise has proved to be effective against obesity [16] and also to maintain or to improve balance in elderly people and in patients with reduced mobility [17].

The whole body vibration (WBV) is a training modality used in elderly and mobility reduced people [18,19]. Although some evidence shows the positive effect of WBV on balance, mobility and body composition in the elderly, its impact remains inconclusive [20]. The main purpose of the present study is to examine the effect of WBV training on SMP30 in postmenopausal women. In addition, we also evaluated the influence of the training on their physical fitness, via 6MWT (6-min walk test) and TUG (time up and go), and on their body composition, fat, lean and bone mass. It could be hypothesized that 12 weeks of WBV training may improve SMP30 levels, physical fitness and body composition in postmenopausal women.

## 2. Materials and Methods

### 2.1. Participants

Eighteen postmenopausal women were invited to participate in the study. The study was approved by the Local Ethics Committee (87/2013), University of Extremadura Commission of Bioethics and Biosafety. The study protocol complied with the Declaration of Helsinki for Human Experimentation.

The protocol was explained to the interested individuals in a meeting. The eligible participants could not have any medical conditions that precluded their ability to complete the training intervention and the fitness tests. Finally, 15 participants consented to participate in the study, and 13 of them (age = 54.3 ± 3.4 years, body mass = 71.5 ± 9.7 kg, height = 162.6 ± 6.0 cm) completed the study and were included in the statistical analysis. All patients gave their written informed consent for participation in the research study two days before starting the training program. On the same day between 8:30 and 9:30 a.m. the pretest blood samples were collected, and one day before the intervention, the body composition, balance, TUG and 6MWT pre-test were performed in this order. Similarly, post-test blood samples were collected two days after the subjects completed the training program, between 8:30 to 9:30 a.m. again, and the next day body composition and physical fitness post-test were measured.

### 2.2. Body Composition

Height and weight were measured using a 780 SECA digital column scale (Hamburg, Germany), which allowed subsequent estimation of the body mass index (BMI). In addition, body composition was assessed using a BC-4188-contact electrode BIA device (Tanita Corp., Tokyo, Japan), following the standard operating procedures based on the manufacturer’s instructions.

### 2.3. Physical Fitness

Gait performances were measured with the 6MWT and the TUG. The 6MWT was performed once and determined the maximum distance, in meters, that a participant can walk as fast as possible in 6 min in a 20 m corridor. After the completion of the test the distance was recorded [21]. The TUG was performed twice and the time was measured in seconds. A subject would rise from a chair, walk 3 m, turn around, walk back to the chair and sit down. The result of best trial was noted [22]. A chronometer was used to record the time taken to complete the test.

Balance was assessed with Biodex Balance System (BBS) (Biodex, Shirley, NY, USA). The clinical test of sensory integration of balance (CTSIB) was performed in four conditions: firm surface with eyes open or closed and unstable surface with eyes open or closed. In all four tests, subjects had to maintain their feet on the platform of the BBS for 30 s with 10 s recovery between tests. The BBS is a circular platform that moves on anterior-posterior and medial lateral axes simultaneously, so the participants have to control their balance and movement degree. The BBS interfaces with software Biodex, 1.08, (Biodex, Inc., Shirley, NY, USA) allowing the degree in each axis and then calculating the sway score. The sway index was used for the statistical analysis.

### 2.4. Blood Extraction Procedure

Blood samples were drawn from the median cubital vein using sterile materials by authorized and qualified personal. Blood was immediately mixed with citric-acid dextrose (ACD) (1/6) and centrifuged for 5 min at 750× *g*, upon which platelet-rich plasma (PRP) was isolated from the erythrocytes pellets. PRP was supplemented with apyrase (40 U/mL) and aspirin (100 µM), and then, the platelets were isolated by centrifuging for 20 min at 350× *g*, as described elsewhere [23]. Isolated platelets were finally lysed by suspending in radioimmunoprecipitation assay buffer (RIPA) buffer, and the samples were immediately stored at −80 °C until needed. Similarly, samples of plasma (250 μL) were mixed with an equal volume of RIPA and stored at −80 °C.

### 2.5. Western Blotting

Samples of either platelet lysates or plasma were unfrozen, and proteins were immediately denaturalized under reducing conditions by mixing with an equal volume of Laemmli’s buffer (2 ×, 10% DTT). Protein-containing samples were heated for 10 min at 70 °C, and subsequently frozen at 4 °C for completing protein denaturalization. Protein samples were finally separated by 10% Sodium Dodecyl Sulfate polyacrylamide gel electrophoresis (SDS)-page. Western Blotting was completed using nitrocellulose membranes and the specific anti-SMP30 antibody diluted 1:1000 in Tris-Buffered Saline, 0.1% Tween^®^ 20 Detergent (TBST) and incubated for 1 h at room temperature. After using the appropriated HRP-conjugated secondary antibody (1:10000 for 1 h) membranes were developed using super-signal solution and a C-Digit device (Li-Cor^®^, Lincoln, NE, USA). Membranes were reprobed with an anti-actin antibody (1:1000 for 1 h) in order to ascertain that similar amounts of protein were loaded in each cell line. The samples were pooled together and loaded in the same gel by intercalating pre-test and post-test.

### 2.6. WBV Training

During the training, the subjects stood on the Galileo 900 Platform (Novotec Medical GmbH, Pforzheim, Germany), holding a half-squat with a 150° knee angle during the exposure to the vibration and relaxed the knee angle during the recovery. They positioned their feet on the mark three around the center of the oscillating platform. The training program lasted 3 months, with 3 sessions per week, the intensity increasing progressively each month, from 12 Hz to 24 Hz, with an amplitude of 3 mm. The working time (duration) and repetitions varied from one to three, and the recovery time was 1 min between repetitions (Table 1).

### 2.7. Statistical Analysis

Descriptive statistics, mean and standard deviation, were calculated for each test. The normality distribution of the data was checked using Shapiro-Wilks test. A paired t-test was used to determine the statistical significance differences between pre-test and post-test. Statistical significance was set at *p* < 0.05. All statistical analyses were performed using IBM SPSS Statistics-v 25.0 for Windows (SPSS, Inc., Chicago, IL, USA).

## 3. Results

### 3.1. SMP30

**Figure 1:**
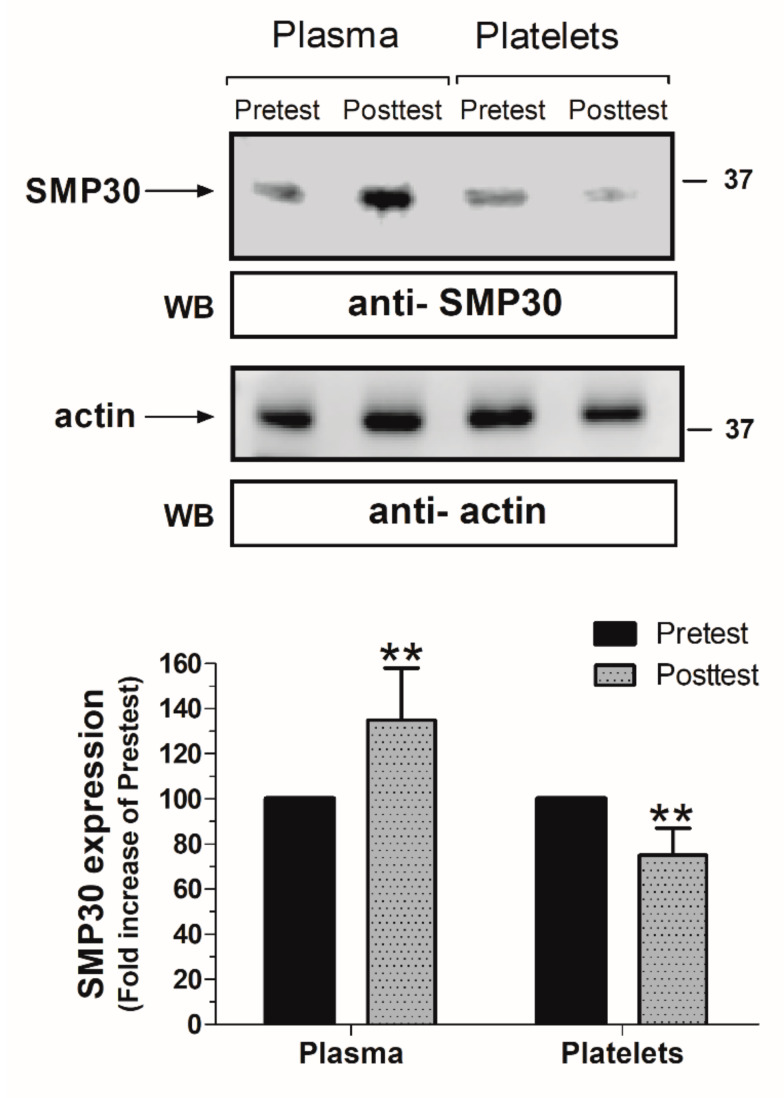
Changes in regucalcin (SMP30) content after the twelve weeks of Whole Body Vibration training programme. ** *p* < 0.01. Significant within-group difference in plasma and platelets.

Western blotting analyses of the blood samples extracted from postmenopausal women before and after three months of WBV revealed differences in the SMP30 concentration. As depicted in Figure 1, the WBV program evoked a reduction of 38.7% in the platelet content of SMP30 (*p* < 0.05; *n* = 13). Meanwhile, we detected an increase of 27.7% in the SMP30 plasma content as a result of the application of the WBV program to the postmenopausal women. This increase in the circulating SMP30 values might be explained due to the cell protein secretion in response to exercise (see also Table 2). 

### 3.2. Physical Fitness

In the postmenopausal population analyzed, WBV was similarly efficient for improving the physical capabilities of the subjects, as demonstrated by the significant reduction of 23.1% found in TUG, as well as by the increase of 12.5% obtained in the 6MWT (Table 2). However, three months WBV training was unable to improve body balance. 

### 3.3. Body Composition

The three months of the WBV program was able to significantly reduce fat mass by 4.4% (*p* < 0.05) compared to baseline data, but other parameters such as bone mass, lean mass, body fat-free mass, and weight, so therefore BMI, remained unaltered in the postmenopausal women (Table 2).

## 4. Discussion

Exercise has been shown to improve body composition, physical condition, and health. However, some debate still remains regarding what is the best type of exercise. While reduced impact exercise might be recommended for certain populations such as elderly people, more vigorous and aerobic exercise such as running is recommended for the middle-aged population in order to maintain fitness. Regarding WBV, many studies reinforce the advantages of this type of training with respect to other programs, and it has been recommended for subjects suffering from certain pathologies such as severe chronic obstructive pulmonary disease [24]. The present study evaluated the effect of three months of WBV in a postmenopausal women population. The 6MWT is a valid test for measuring the functional capacity of elderly people [25] and has been used to evaluate physical capacity in postmenopausal women [26]. It has been shown that patients with osteopenia scored significantly lower on physical capacity (6MWT) compared to the matched controls [26]. Knowing that sedentary behavior during aging accentuates negative physiological effects, one of these is sarcopenia and as a consequence there is a decline in muscle strength. It is known that a decline in muscle strength can affect walking speed negatively [27]. So, the WBV training was effective in maintaining or improving physical capacity in elderly people. Our results in the 6MWT (589 m) is similar to other studies with postmenopausal women (569 m) [28] However, they did not improve the 6MWT performance after three months of muscle strengthening of the lower limbs, while we observed an improvement of 74 m (12.5%), which could be explained by the differences in the training programs between the studies.

WBV training also improved performance in the TUG test, a reliable and valid test for quantifying functional mobility in elderly people [22]. In our results, the improvement of 23.1% is in agreement with previous studies that also showed improvement of 6.2% in TUG time performance after three months of exercise in postmenopausal women with osteoporosis [29]. 

We did not observe any improvement in balance after three months of training using the CTSIB. Contrarily, some studies found that balance can be improved (29%) after eight months of WBV training using the flamingo test [30], after six weeks of vibration training plus physical therapy using the Tinetti test with an improvement of 3.5 ± 2.1 points on the body balance [31], or after six weeks of WBV using the Tinetti with an improvement of 3.7% [32]. Some studies did not find an improvement in balance using the BBS after eight months of WBV training [33]. In this study the balance test used was different from the CTSIB test used in our study. The CTSIB has been used previously to show difference in balance performance between postmenopausal women with or without fibromyalgia [34]. Different balance tests or training programs could explain the discrepancies between studies.

With respect to body composition, we observed a significant fat decrease after the WBV program in postmenopausal women, which is in concordance with previous studies [35]. These authors observed a decrease in body fat (−2.2%) after three months of WBV training. In our study the decrease was −4.4%. This might be explained by the changes found in the content of the SMP30 in the plasma of women, since SMP30 was reported to participate in the regulation of lipid by the liver [36]. As previously described, SMP30 participates in lipid metabolism due to its regulatory role on the early stage of insulin production, and it has been proposed as a key factor in impaired regulation of glucose in aging [37,38].

Hormonal changes were already reported in older people by applying a unique WBV session lasting for 5 min, the changes found in IGF-1 and cortisol being particularly relevant. Both are relevant for fat metabolism at either the liver or visceral fat [39,40]. However, no experimental evidence exists regarding the possible relationship between circulating SMP30 levels and IGF-1, despite that both were found elevated in response to WBV, and have been involved in liver tissue structure and lipid metabolism by the liver, which deserves future research [41,42,43].

Regarding bone structure and composition, we were unable to detect significant changes in the bone content of postmenopausal women after twelve weeks of WBV, which reinforces previous data available in the literature, where the authors observed no effect of WBV on bone mineral density in postmenopausal women and other populations [20,33,44]. On the contrary, previous studies done by us and other groups claimed a positive effect of WBV on bone mineralization [30,45,46]. The discrepancy could be explained by the different time period and intensity of the training programs used, but also the methodology used for monitoring bone mass [46].

On the other hand, a regulatory role of SMP30 on osteoblastic and osteoclastic cells models has been proposed [47]. In fact, overexpression of exogenous SMP30 in mice resulted in bone weakness, similar to the osteoporosis process [48]. Thus, the discrepancy in bone mineral content found due to WBV may be explained by the fact that a prolonged time period of WVB training might deregulate the values of intracellular and circulating SMP30. In addition, protein platelet content has previously been used as marker cells during the appearance and progression of several pathologies of different nature, such as Alzheimer’s and cancer [49,50]. So it could be possible that WBV would increase the plasma concentration of SMP30, by inducing their hyperproduction from the liver and kidney, but also by mobilizing cell deposits, as evidenced by the SMP30 mobilization from platelets in the WBV program demonstrated here. SMP30 secretion by the cells would decrease the intracellular content, so its function could revert in the osteoclastic and osteoblastic cells, which would lead to bone mineralization, but future research will be required to conclude the existence of such a mechanism.

A limitation of our study is that there was no control group. It should be interesting to compare the effect of a training group with another group without intervention. However, as we described before, it is a pilot study whose primary outcome was to analyze the effect of training on human levels of SMP30. Further research is recommended to better understand the effect of any kind of training on SMP30. Although the present study would gain more information if we could arrange intermediate testing points, we decided to perform only blood, body composition and physical fitness tests at the beginning and at the end of the training program, to avoid unnecessary suffering to the subjects which is in agreement with the ethical guidelines. That we observed significant changes in SMP30 after the exercise could be interesting for future studies to evaluate the changes, in a time-dependent curve, in the expression of SMP30 under this type of training programs. Another limitation was that diet and physical activity were not controlled, but subjects were encouraged to maintain their usual physical activities and nutritional status. Knowing that all subjects lived in a nursing home where habits and customs were the same, not much change i diet or physical activity could occur from baseline to follow-up.

## 5. Conclusions

In conclusion, the present study suggests that twelve weeks of WBV training, three times per week, enhances circulating SMP30, gait performance and reduce fat mass in postmenopausal women.

## Figures and Tables

**Table 1:** The Whole Body Vibration training program.

Traininig Variables	Traininig Characteristics per Week											
Weeks	1	2	3	4	5	6	7	8	9	10	11	12
Frequency (Hz)	12	12	12	12	18	18	18	18	24	24	24	24
Amplitude (mm)	3	3	3	3	3	3	3	3	3	3	3	3
Duration (min)	1	1	1	1	2	2	2	2	3	3	3	3
Repetitions	1	1	1	1	2	2	2	2	3	3	3	3
Recovery (min)	1	1	1	1	1	1	1	1	1	1	1	1

**Table 2:** SMP30, physical fitness and body composition before (pre-test) and after (post-test) the Whole Body Vibration training program. Mean ± standard deviation.

Variables	Pretest			Posttest			Effect Size
SMP30 in Plasma (A.U.)	699.7	±	192.4	893.7	±	223.1 **	0.93
SMP30 in Platelets (A.U.)	1313.4	±	479.3	805.5	±	307.4 **	−1.26
TUG (s)	7.8	±	0.8	6	±	0.4 *	−2.85
6MWT (m)	589.2	±	35.5	663.1	±	38.2 **	2.0
Fat mass (%)	27.5	±	6.2	26.3	±	5.7 *	−0.20
Bone mass (%)	2.2	±	0.3	2.2	±	0.2	0
Lean mass (%)	41.9	±	3.9	42.0	±	4.2	0.03
Body fat-free mass (%)	43.9	±	4.1	44.2	±	4.4	0.07
Weight (kg)	71.5	±	9.7	70.5	±	9.5	−0.10
BMI (kg/m^2^)	27.0	±	3.1	26.6	±	3.0	−0.13
Balance FSEO (sway index)	0.68	±	0.16	0.59	±	0.31	−0.37
Balance FSEC (sway index)	0.74	±	0.26	0.83	±	0.31	0.32
Balance USEO (sway index)	0.83	±	0.32	0.72	±	0.38	−0.31
Balance USEC (sway index)	0.74	±	0.23	0.80	±	0.41	0.18

SMP30: Regucalcin; A.U.: Arbitrary units; TUG: Time up and go; 6MWT: 6-min walk test; BMI: Body mass index; FSEO: Firm surface eyes open; FSEC: Firm surface eyes closed; USEO: Unstable surface eyes open; USEC: Unstable surface eyes closed. * *p* < 0.05; ** *p* < 0.01.
